# Supplementary figures and images for: Fourier-transform infrared spectroscopy for typing of vancomycin-resistant Enterococcus faecium: performance analysis and outbreak investigation
Source: Microbiol Spectr. 2023 Sep 22;11(5):e00984-23. doi: 10.1128/spectrum.00984-23 (PMC10581122; doi:10.1128/spectrum.00984-23)

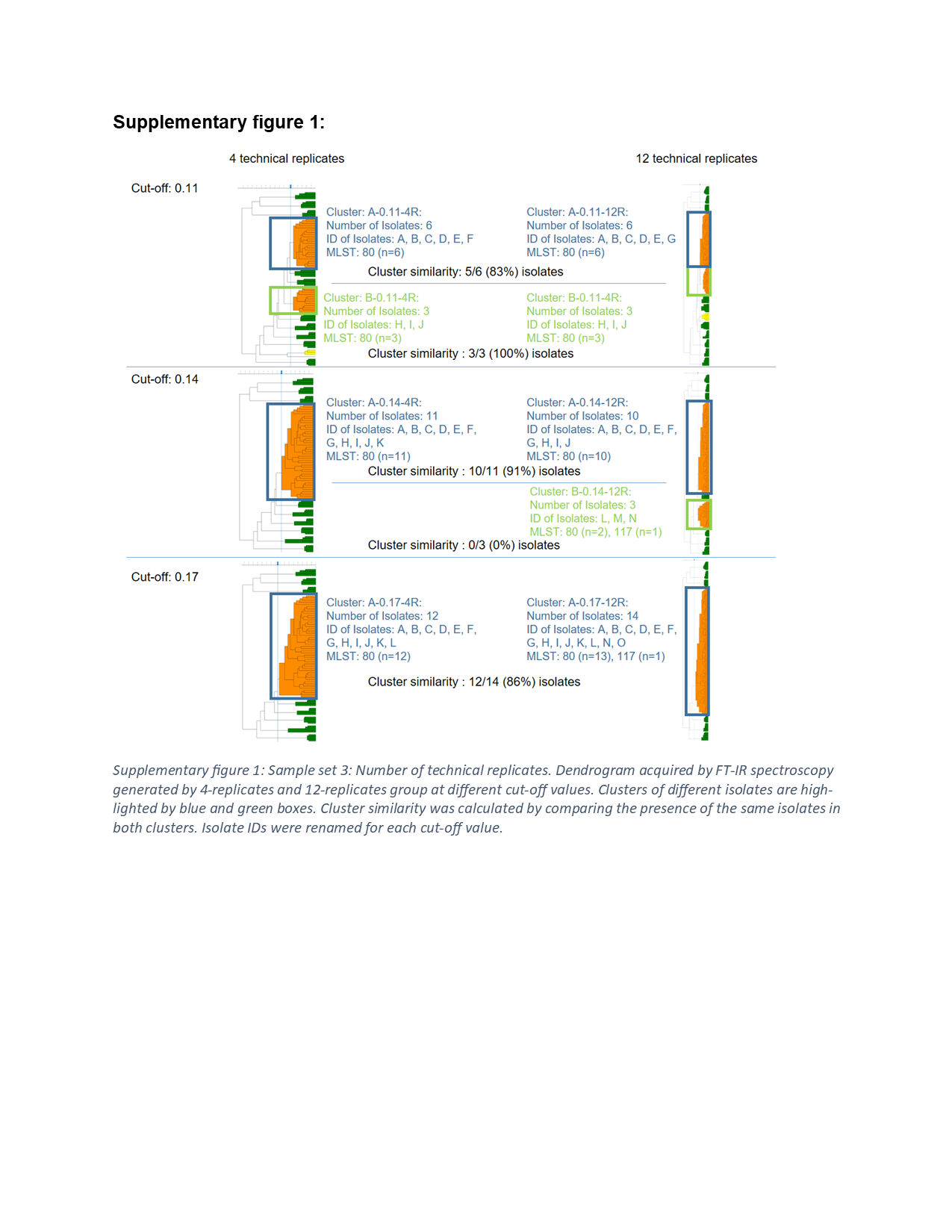

Supplement: Figure S1 — Sample set 3: Number of technical replicates. [file spectrum.00984-23-s0001.tif]

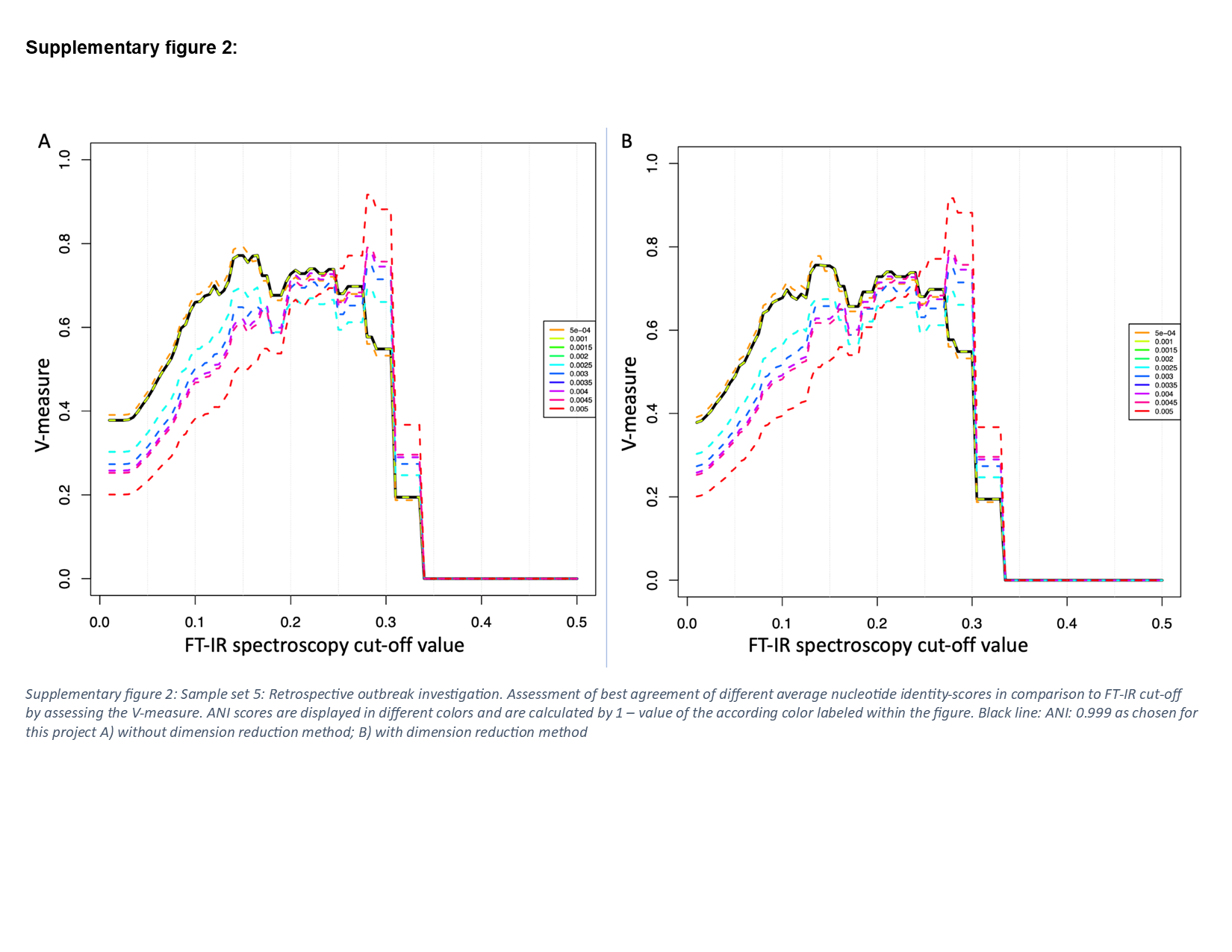

Supplement: Figure S2 — Sample set 5: Assessment of best agreement. [file spectrum.00984-23-s0002.tif]

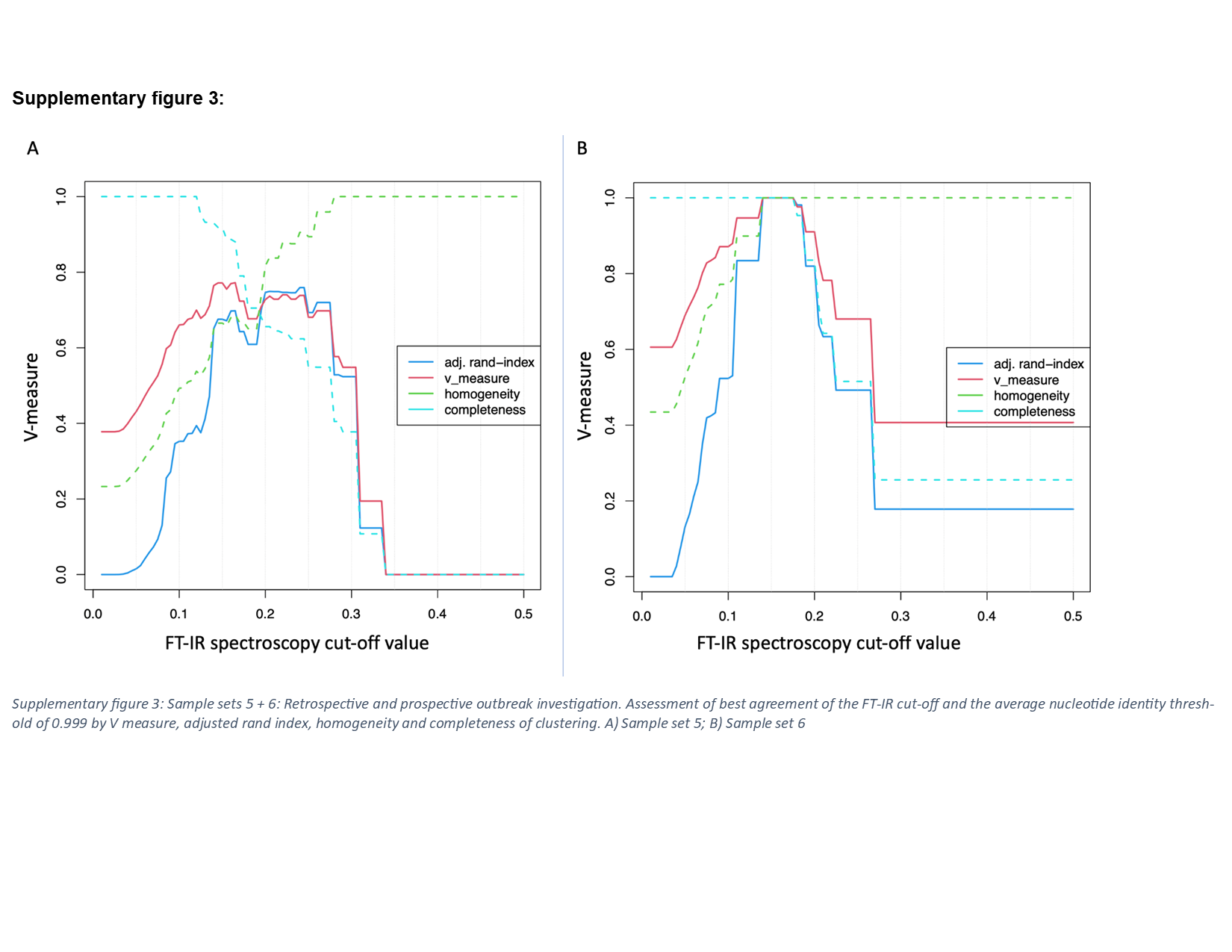

Supplement: Figure S3 — Sample set 5+6: Assessment of best agreement. [file spectrum.00984-23-s0003.tif]

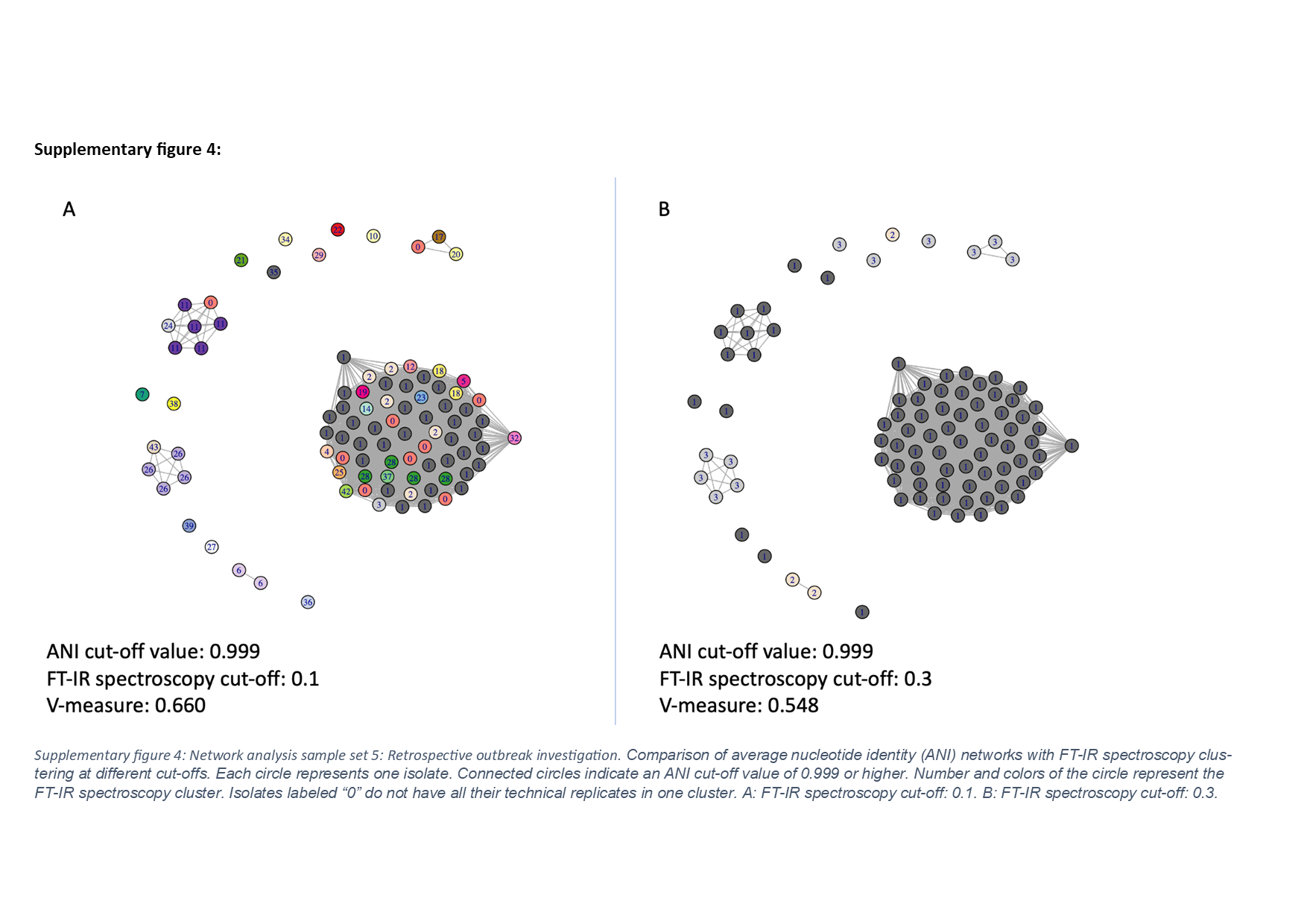

Supplement: Figure S4 — Sample set 5: Comparison of ANI networks to FT-IR. [file spectrum.00984-23-s0004.tif]

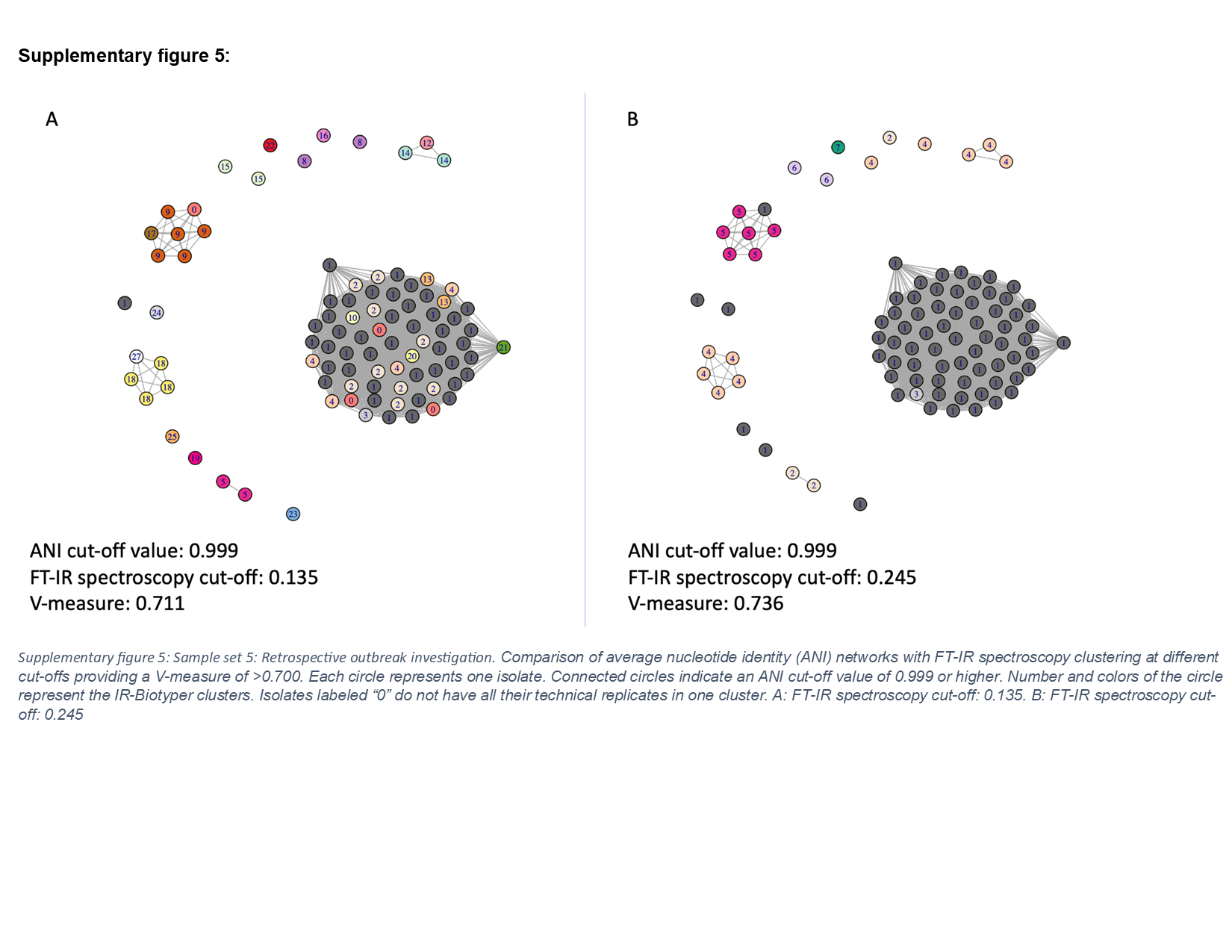

Supplement: Figure S5 — Sample set 5: Comparison of average nucleotide identity (ANI) networks with FT-IR spectroscopy clustering at different cut-offs providing a V-measure of >0.700. [file spectrum.00984-23-s0005.tif]

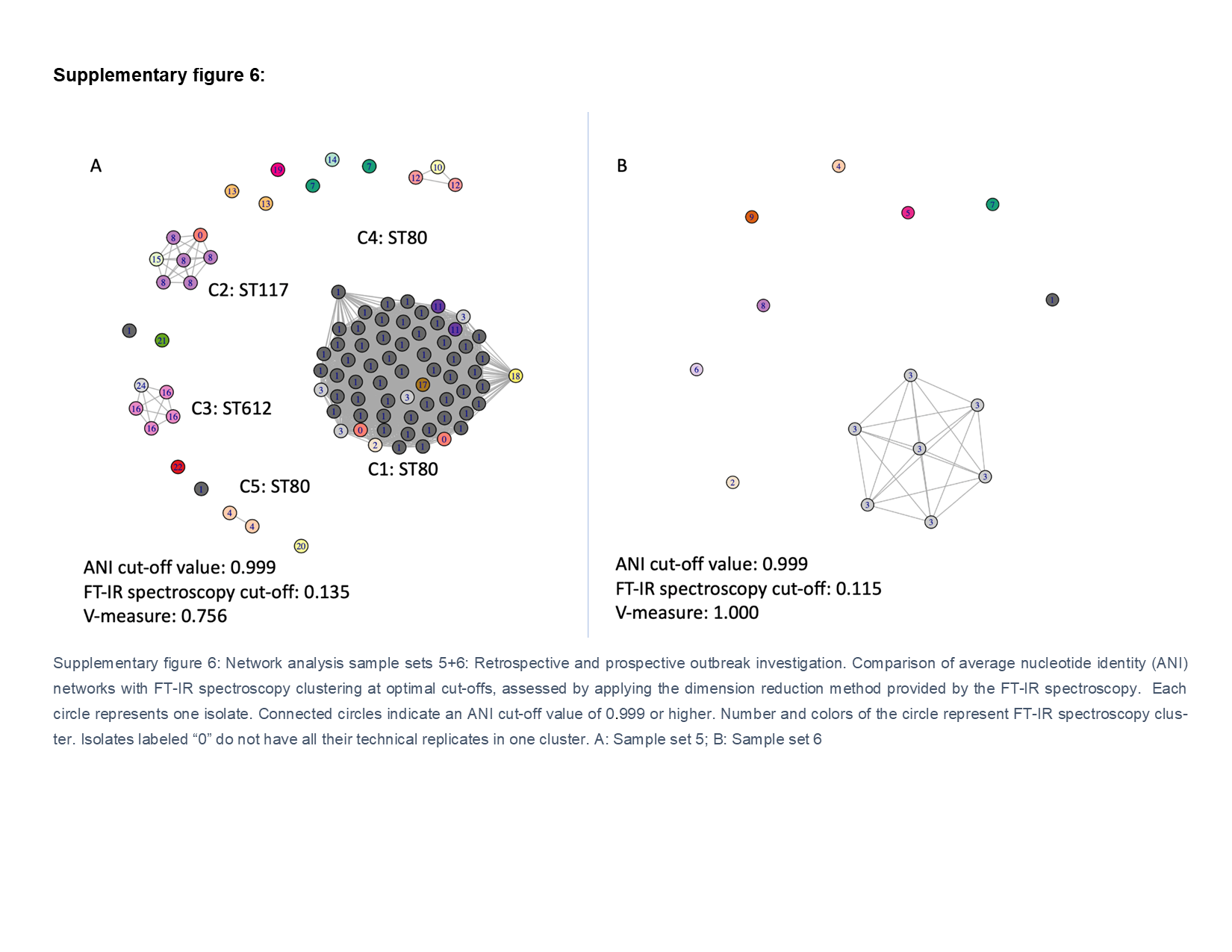

Supplement: Figure S6 — Sample set 5+6: Comparison of average nucleotide identity (ANI) networks with FT-IR spectroscopy clustering at optimal cut-offs. [file spectrum.00984-23-s0006.tif]

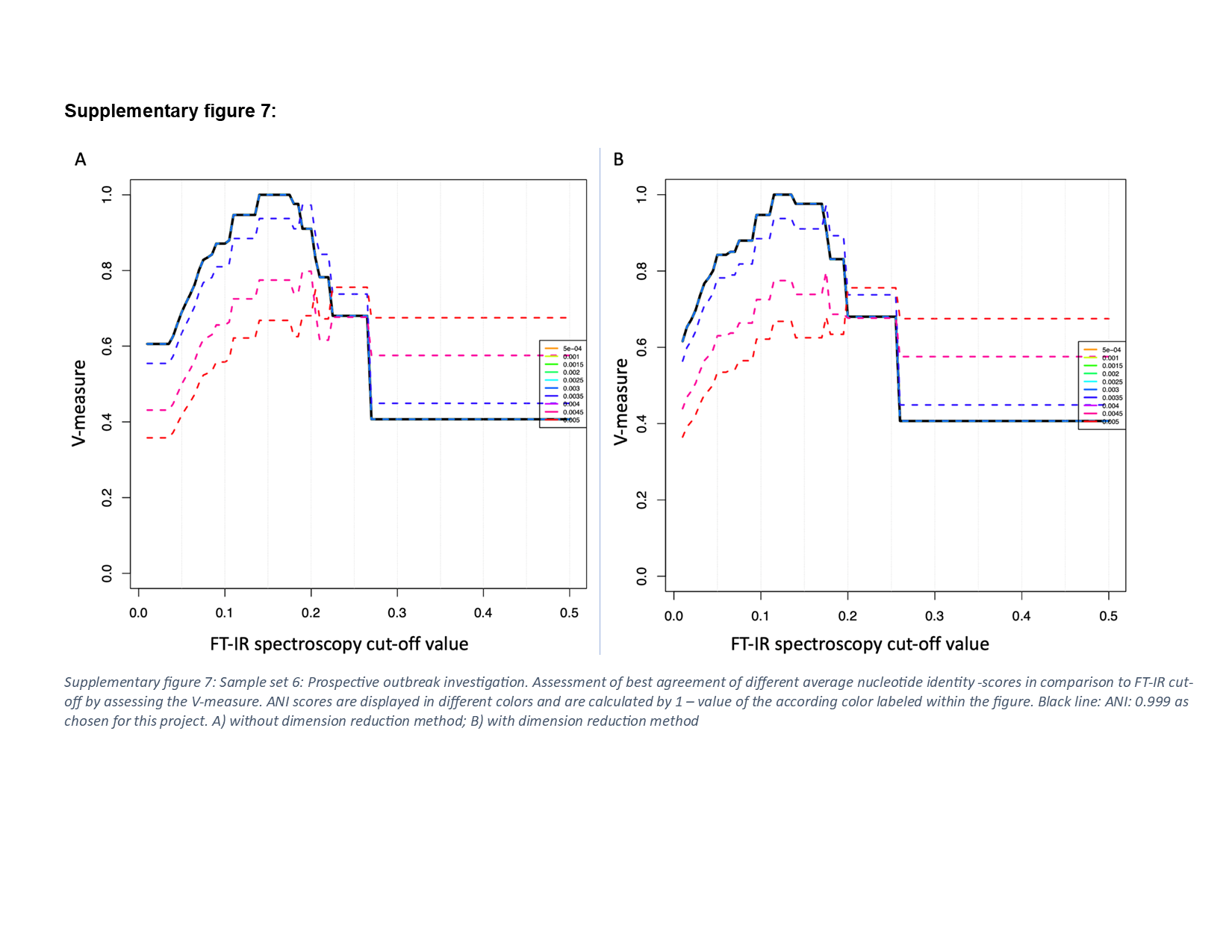

Supplement: Figure S7 — Sample set 6: Assessment of best agreement. [file spectrum.00984-23-s0007.tif]

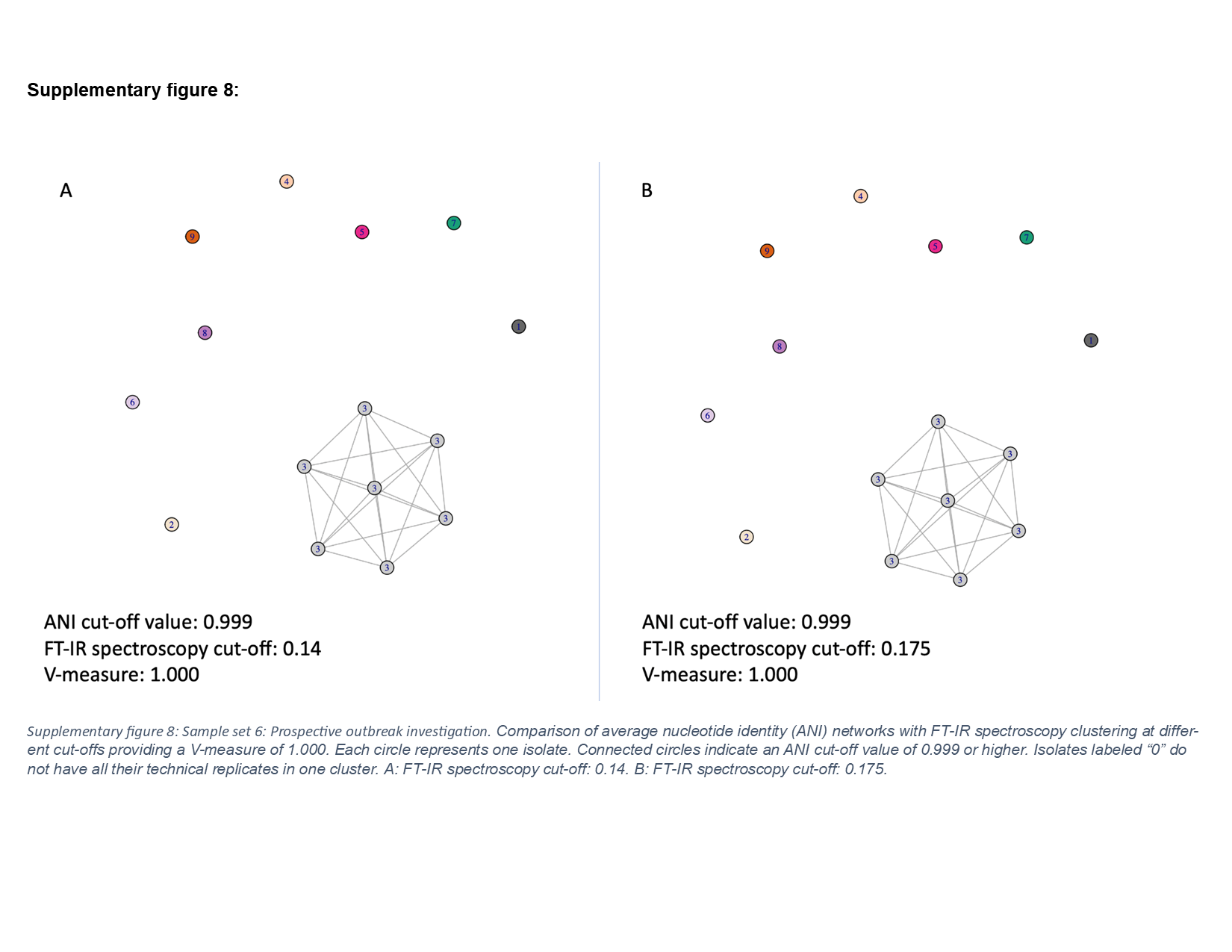

Supplement: Figure S8 — Sample set 6: Comparison of average nucleotide identity (ANI) networks with FT-IR spectroscopy clustering at different cut-offs providing a V-measure of 1.000. [file spectrum.00984-23-s0008.tif]

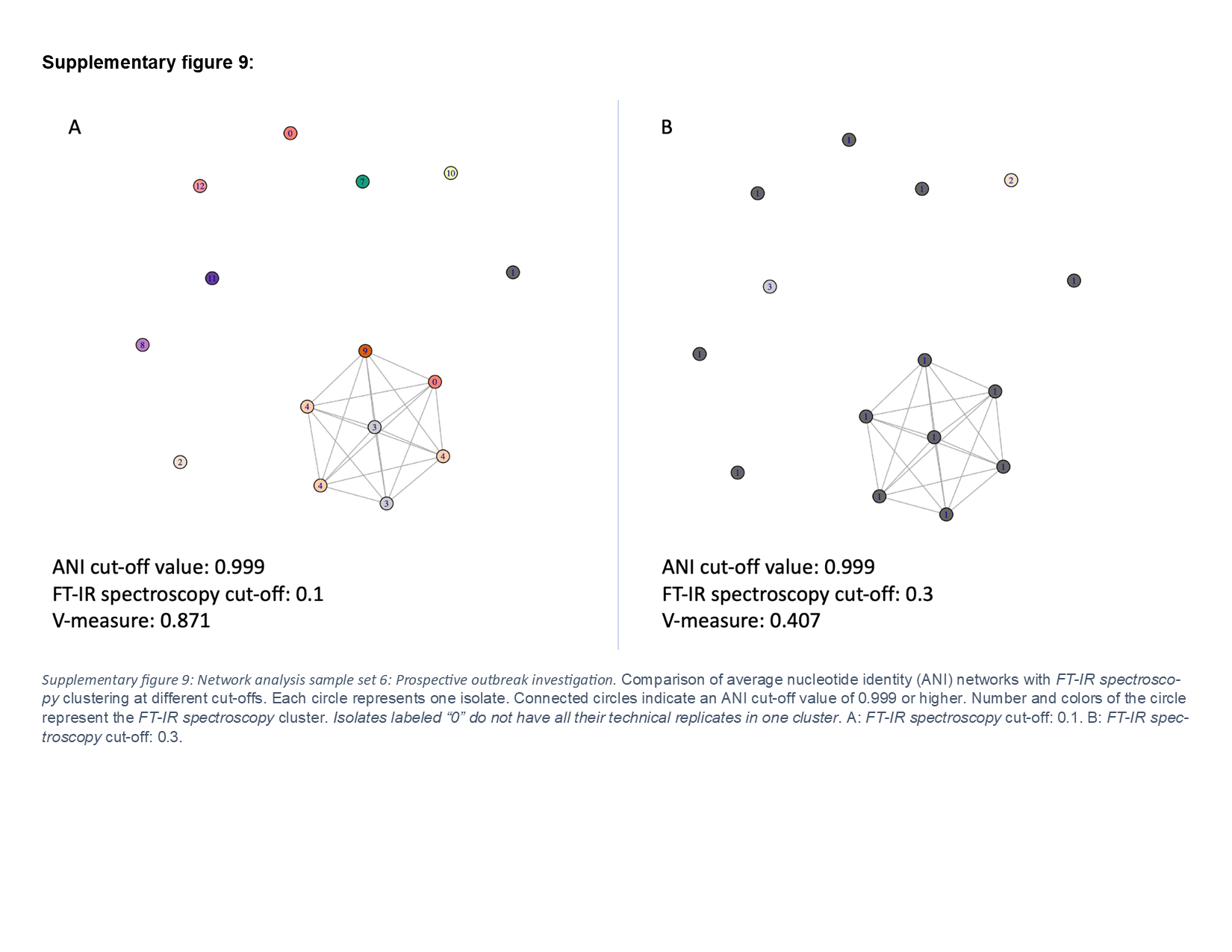

Supplement: Figure S9 — Sample set 6: Comparison of average nucleotide identity (ANI) networks with FT-IR spectroscopy clustering at different cut-offs. [file spectrum.00984-23-s0009.tif]
